# Supplementary material for: Structural basis of ion transport and inhibition in ferroportin
Source: Nat Commun. 2020 Nov 10;11:5686. doi: 10.1038/s41467-020-19458-6 (PMC7655804; doi:10.1038/s41467-020-19458-6)
Supplement: Supplementary file 1 — Supplementary Information [file 41467_2020_19458_MOESM1_ESM.pdf]

Supplementary Information

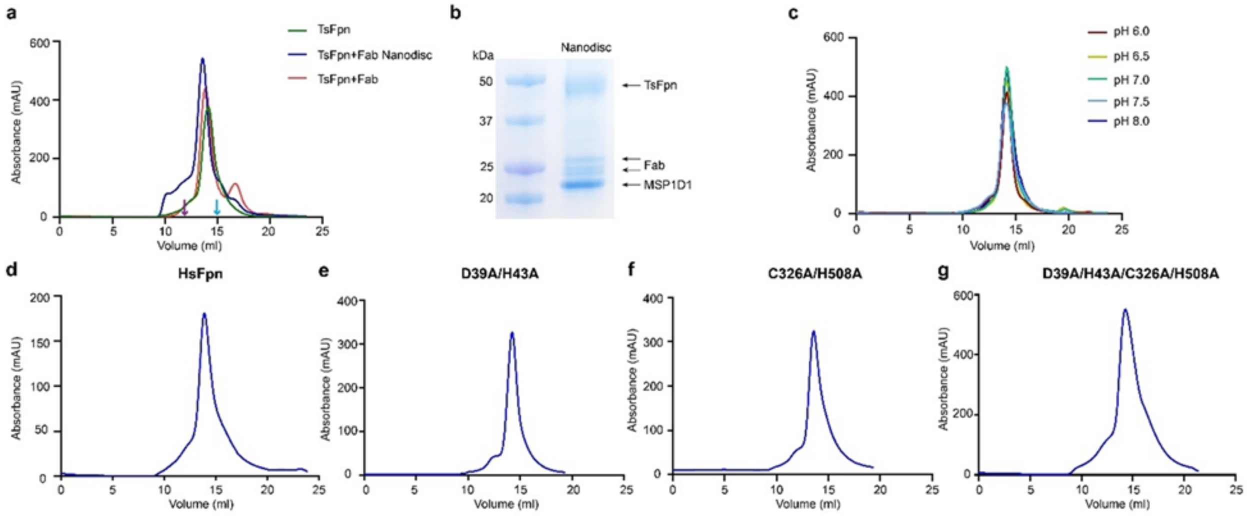

Supplementary Figure 1. Wild type and mutant TsFpn proteins. a. Size-exclusion chromatography of TsFpn in complex with Fab of 11F9 before and after reconstitution into nanodiscs. Elution volumes of membrane proteins of known molecular weight, bcMalT (100 kDa, orange)[1] and mouse SCD1 (41 kDa, blue)[2] are marked by arrows. **b.** SDS-PAGE of the reconstitution. TsFpn runs as a diffused band on the gel likely because it is glycosylated. **c.** Size-exclusion chromatography of TsFpn in pH ranging from 6.0 to 8.0. **d-g.** Size-exclusion chromatography of human Fpn (**d**) and TsFpn with the S1 (Asp39Ala/His43Ala) (**e**), S2 (Cys326Ala/His508Ala) (**f**) and S1+S2 (Asp39Ala/His43Ala/Cys326Ala/His508Ala) (**g**) mutations.

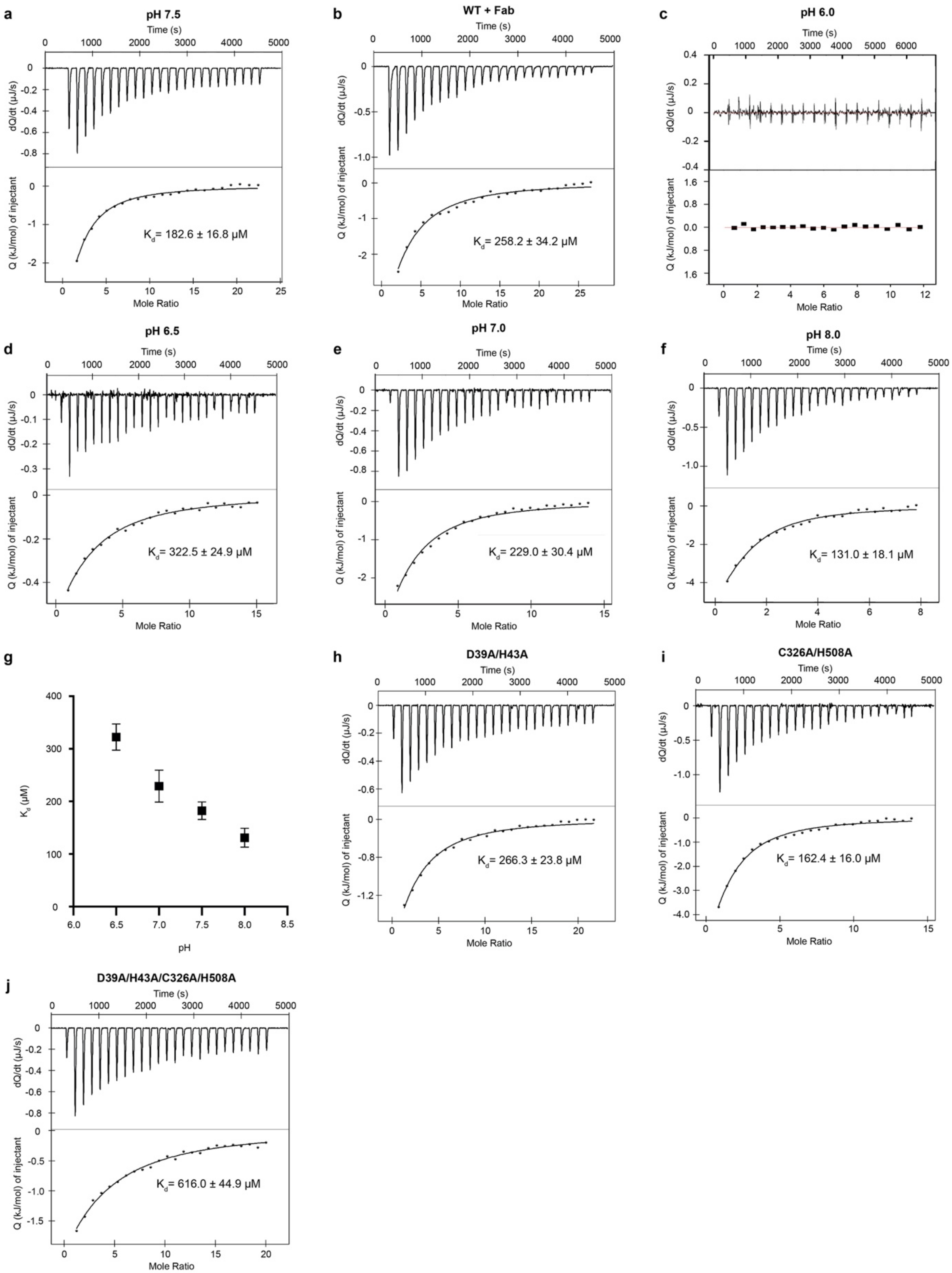

a
pH 7.5
Time (s)
dQ/dt (μJ/s)
Q (kJ/mol) of injectant
K$_d$= 182.6 ± 16.8 μM
Mole Ratio
b
WT + Fab
Time (s)
dQ/dt (μJ/s)
Q (kJ/mol) of injectant
K$_d$= 258.2 ± 34.2 μM
Mole Ratio
c
pH 6.0
Time (s)
dQ/dt (μJ/s)
Q (kJ/mol) of injectant
Mole Ratio
d
pH 6.5
Time (s)
dQ/dt (μJ/s)
Q (kJ/mol) of injectant
K$_d$= 322.5 ± 24.9 μM
Mole Ratio
e
pH 7.0
Time (s)
dQ/dt (μJ/s)
Q (kJ/mol) of injectant
K$_d$= 229.0 ± 30.4 μM
Mole Ratio
f
pH 8.0
Time (s)
dQ/dt (μJ/s)
Q (kJ/mol) of injectant
K$_d$= 131.0 ± 18.1 μM
Mole Ratio
g
K$_d$ (μM)
pH
h
D39A/H43A
Time (s)
dQ/dt (μJ/s)
Q (kJ/mol) of injectant
K$_d$= 266.3 ± 23.8 μM
Mole Ratio
i
C326A/H508A
Time (s)
dQ/dt (μJ/s)
Q (kJ/mol) of injectant
K$_d$= 162.4 ± 16.0 μM
Mole Ratio
j
D39A/H43A/C326A/H508A
Time (s)
dQ/dt (μJ/s)
Q (kJ/mol) of injectant
K$_d$= 616.0 ± 44.9 μM
Mole Ratio

Supplementary Figure 2. ITC measurements of Co^{2+} binding. ITC measurements of Co^{2+} binding to TsFpn (**a**) and TsFpn-11F9 Fab complex (**b**) at pH 7.5. Co^{2+} binding to TsFpn at pH 6.5 (**c**), pH 7.0 (**d**), pH 7.5 (**e**), and pH 8.0 (**f**). **g**. K_d of Co^{2+} binding to TsFpn versus different pH. **h-j**. ITC measurements of Co^{2+} binding to the S1 (**h**), S2 (**i**) and S1+S2 (**j**) mutant TsFpn. In each graph, the top panel is the rate of heat release versus time and the bottom panel is heat from each injection versus the molar ratios of Co^{2+} and TsFpn. Data points are fit with a single-binding site equation to extract the K_d.

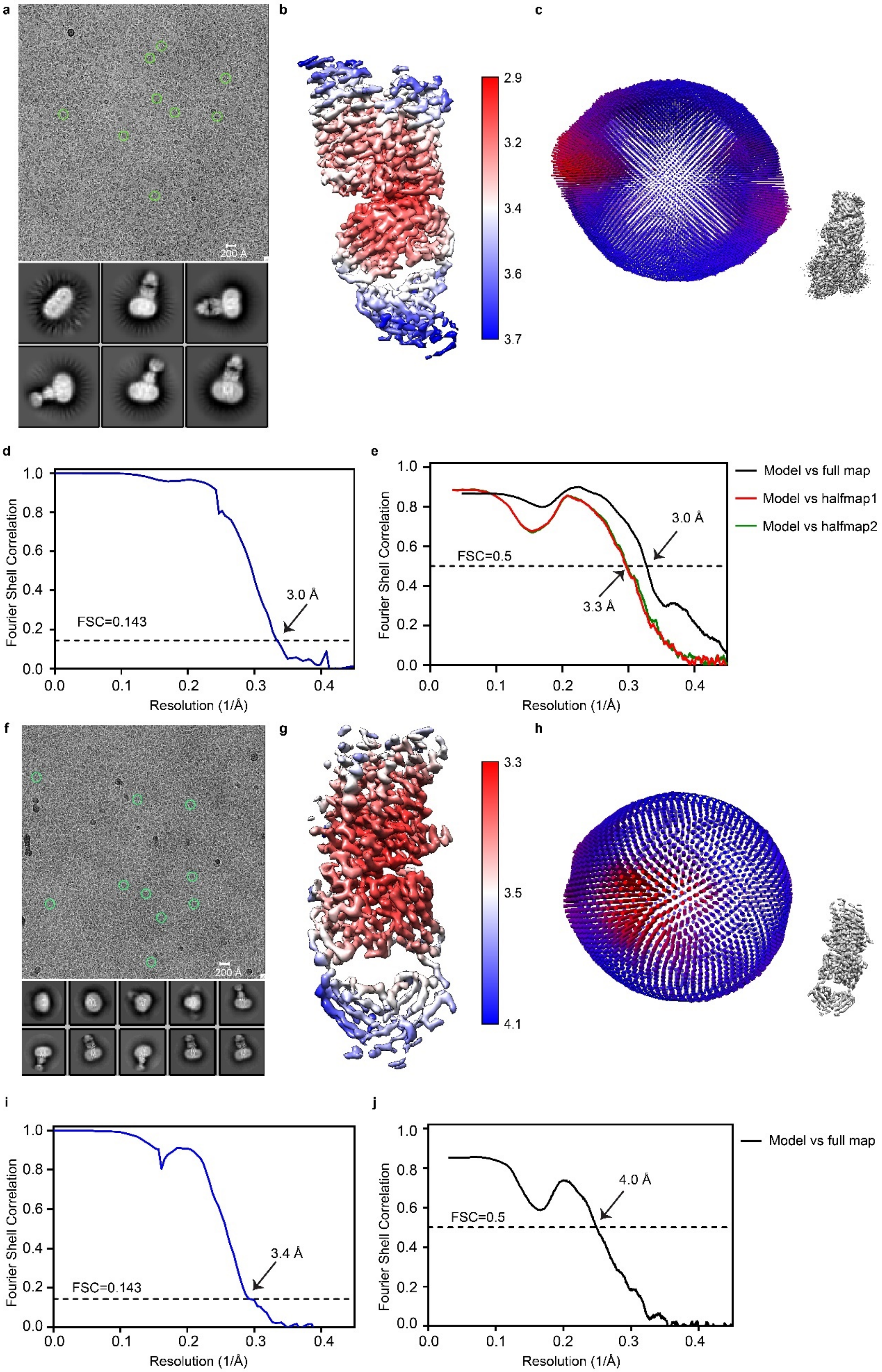
a
b
c
200 Å
2.9
3.2
3.4
3.6
3.7
d
e
1.0
0.8
0.6
0.4
0.2
0.0
Fourier Shell Correlation
FSC=0.143
3.0 Å
0.0 0.1 0.2 0.3 0.4
Resolution (1/Å)
Model vs full map
Model vs halfmap1
Model vs halfmap2
FSC=0.5
3.0 Å
3.3 Å
f
g
h
200 Å
3.3
3.5
4.1
i
j
FSC=0.143
3.4 Å
Model vs full map
FSC=0.5
4.0 Å

Supplementary Figure 3. Cryo-EM analysis of the TsFpn-Fab complex reconstituted in nanodiscs. a. Representative electron micrograph and 2D class averages of TsFpn-Fab particle images. **b.** Local resolution map for the 3D reconstruction of the TsFpn-Fab complex. **c.** Euler angle distribution of the TsFpn-Fab complex in the final 3D reconstruction. **d.** The gold-standard Fourier shell correlation (FSC) curve for the final map. **e.** FSC curve of the refined model of the TsFpn-Fab complex versus the full map (black) and individual half maps (red and green). **f.** Representative electron micrograph and 2D class averages of TsFpn-Fab-hepcidin particle images. **g.** Local resolution map for the 3D reconstruction of the TsFpn-Fab-hepcidin complex. **h.** Euler angle distribution of the TsFpn-Fab-hepcidin complex in the final 3D reconstruction. **i.** The gold-standard FSC curve for the final map of the TsFpn-Fab-hepcidin complex. **j.** FSC curve of the refined model of the TsFpn-Fab-hepcidin complex versus the full map (black).

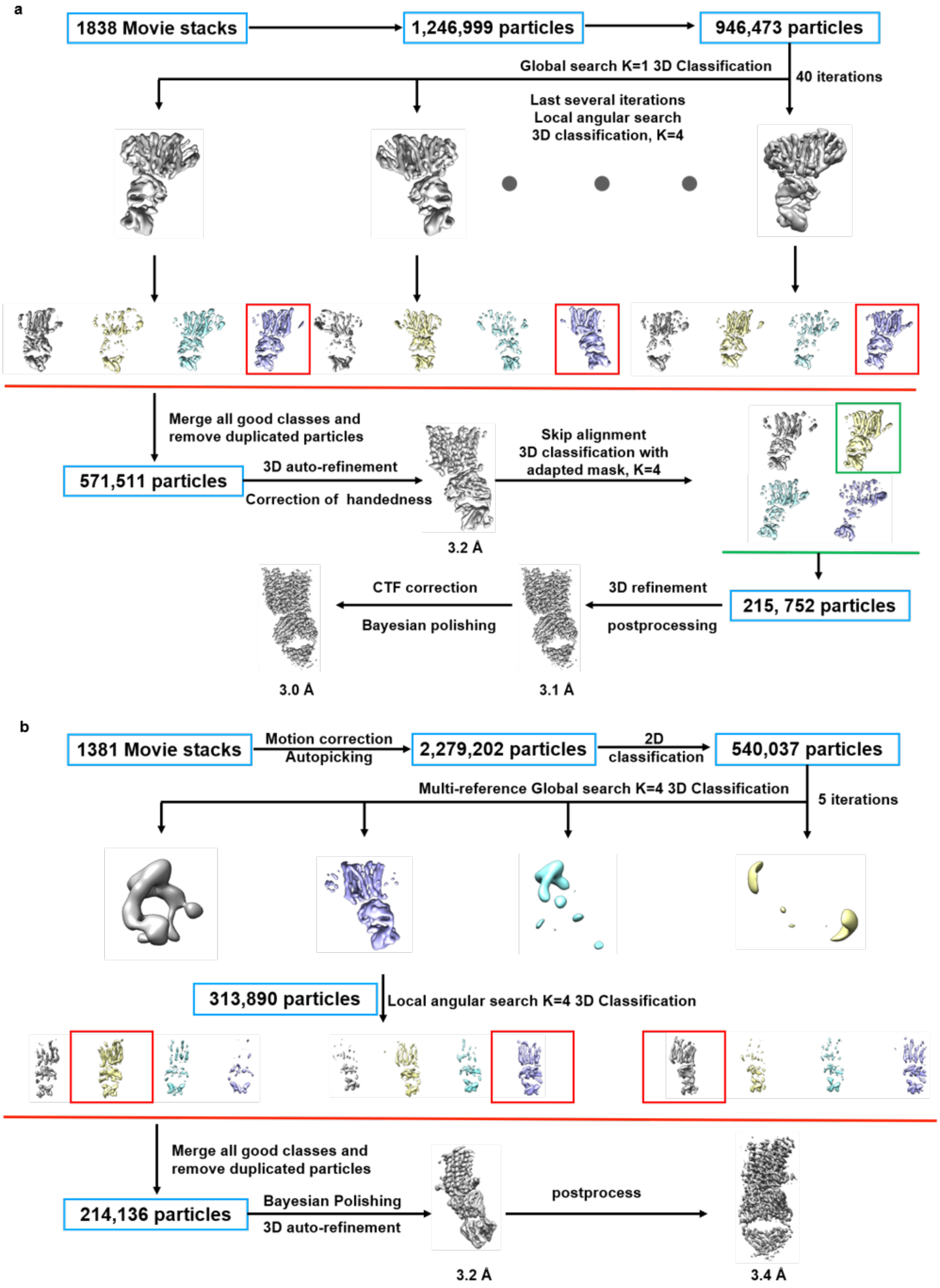
a
1838 Movie stacks
1,246,999 particles
946,473 particles
Global search K=1 3D Classification
40 iterations
Last several iterations
Local angular search
3D classification, K=4
Merge all good classes and
remove duplicated particles
571,511 particles
3D auto-refinement
Correction of handedness
3.2 Å
Skip alignment
3D classification with
adapted mask, K=4
215, 752 particles
3D refinement
postprocessing
3.1 Å
CTF correction
Bayesian polishing
3.0 Å
b
1381 Movie stacks
Motion correction
Autopicking
2,279,202 particles
2D
classification
540,037 particles
Multi-reference Global search K=4 3D Classification
5 iterations
313,890 particles
Local angular search K=4 3D Classification
Merge all good classes and
remove duplicated particles
214,136 particles
Bayesian Polishing
3D auto-refinement
3.2 Å
postprocess
3.4 Å

Supplementary Figure 4. Flow chart of Cryo-EM data processing of TsFpn-Fab in the presence of Co^{2+} (a) and hepcidin (b).

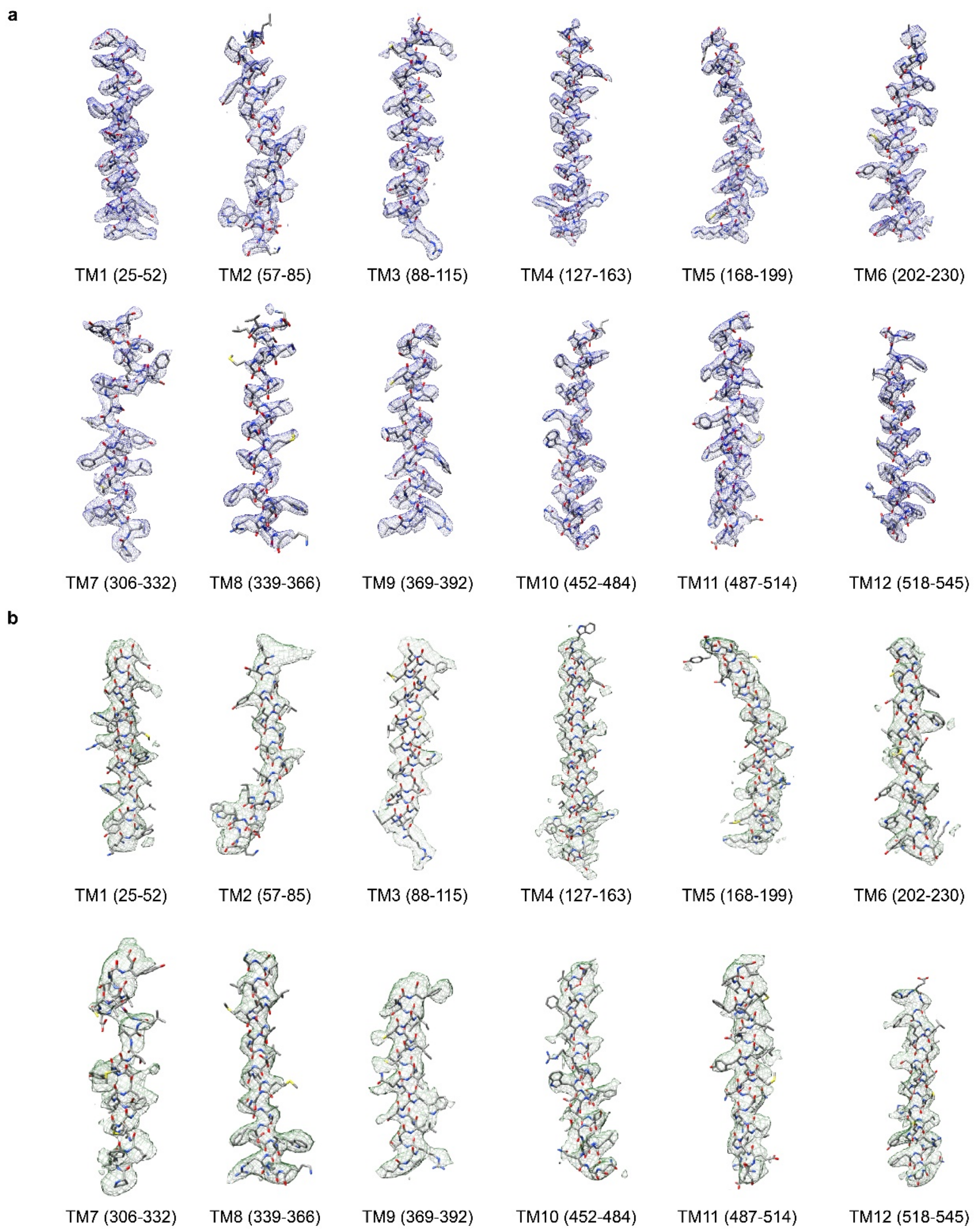

Supplementary Figure 5. Representative densities of transmembrane helices in TsFpn-Fab in the presence of Co^{2+} (a) and hepcidin (b).

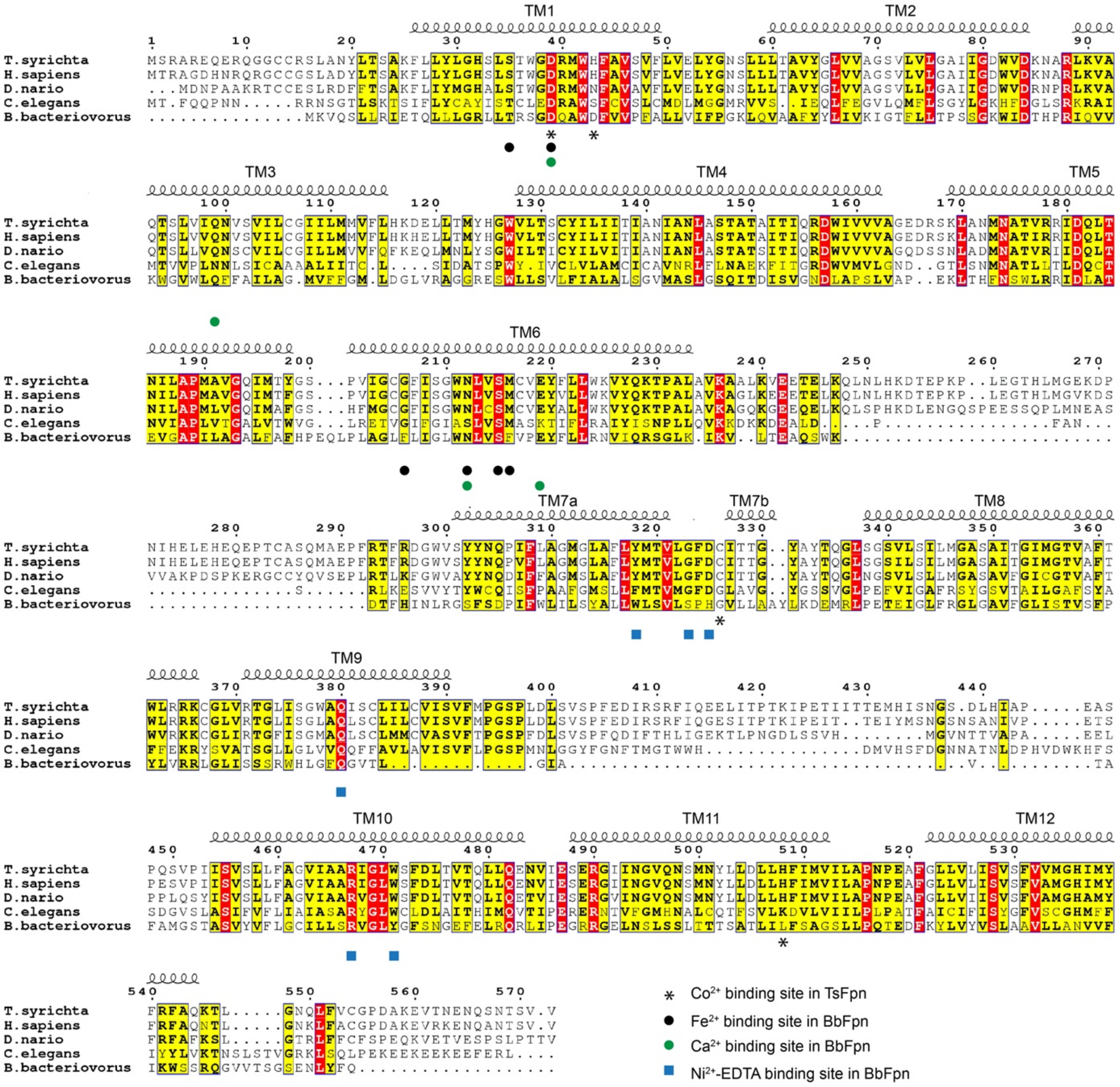

Supplementary Figure 6. Fpn sequence alignment. Fpn from Philippine tarsier (Uniprot accession number A0A1U7U6F1), human (Q9NP59), zebrafish (Q9I9R3), worm (Q8IA95), and BbFpn (Q6MLJ0) are aligned using the Clustal Omega server[3]. Secondary structural elements of Fpn are marked above the alignment. Residues are colored based on their conservation using the ESPript server[4]. Residues at the two Co^{2+} binding sites in TsFpn, Fe^{2+}, Ca^{2+} and Ni^{2+}-EDTA binding site in BbFpn are labeled with black asterisks, black circles, green circles and blue squares, respectively.

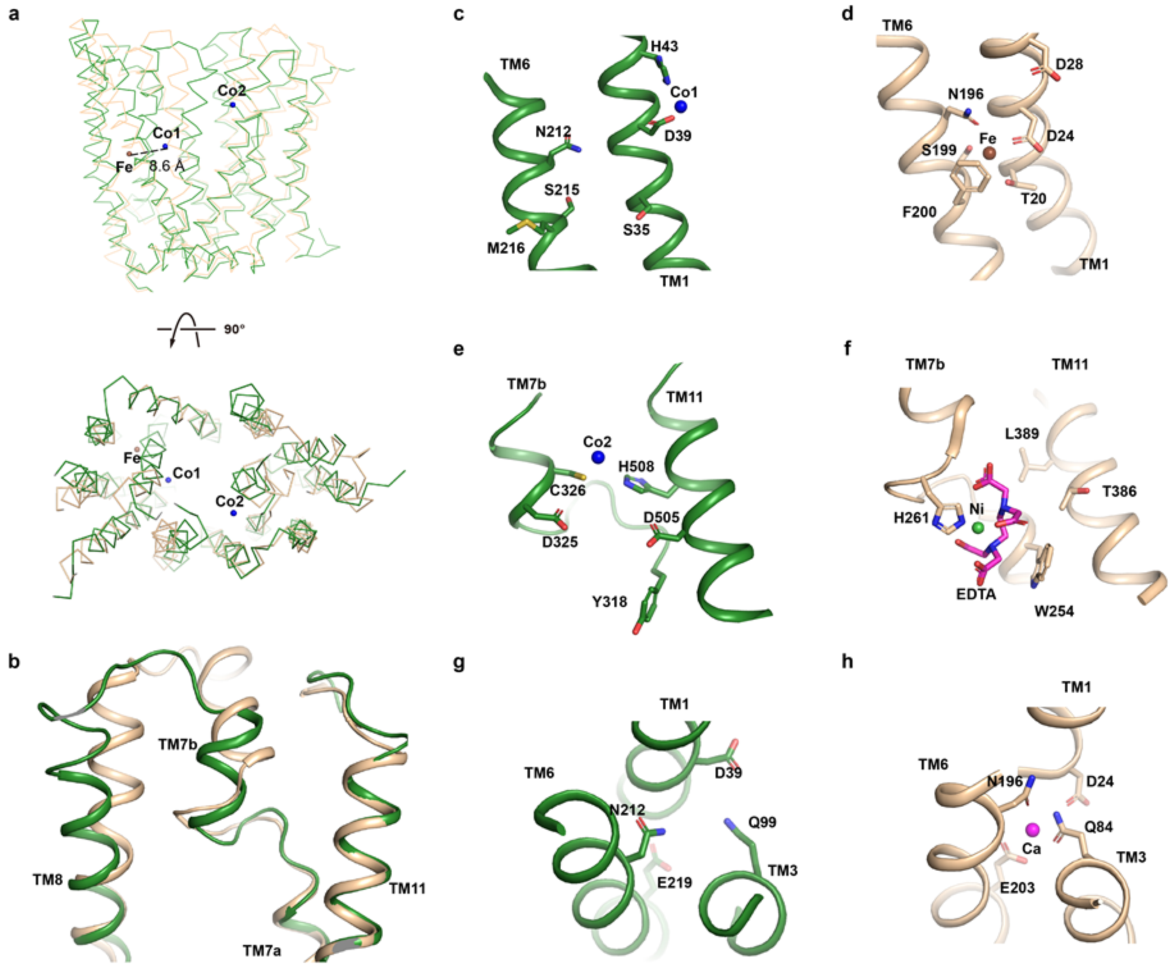

Supplementary Figure 7. Comparison of ion binding sites in TsFpn and BbFpn. a. TsFpn (ribbon in green) is superposed onto BbFpn (ribbon in wheat, PDB ID 5AYM) in two views. Distance between Co1 in TsFpn and Fe in BbFpn is indicated. **b.** Conformational changes in TM7b between TsFpn (cartoon in green) and BbFpn (cartoon in wheat). **c-h.** Metal ion binding sites in TsFpn (**c, e and g**) and BbFpn (**d, f and h**). Side chains of residues participating in binding are shown as sticks.

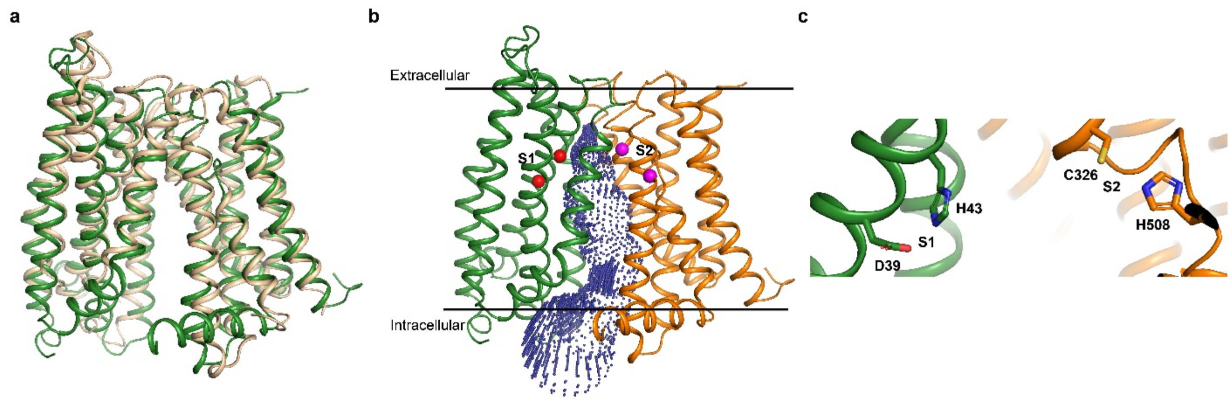

Supplementary Figure 8. Inward-facing model of TsFpn. a. The inward-facing model of TsFpn (cartoon in green) is superposed onto BbFpn (cartoon in wheat, PDB ID 5AYO). **b.** The cartoon representation of the inward-facing model of TsFpn with the N- and C-domains shown in green and orange, respectively. The solvent accessible regions in the cavity, calculated by HOLE[5], is shown as blue dots. C-alphas of residues composing S1 and S2 are shown as spheres and colored red and magenta, respectively. **c.** Close view of S1 and S2 in the inward-facing model with side chains shown as sticks.

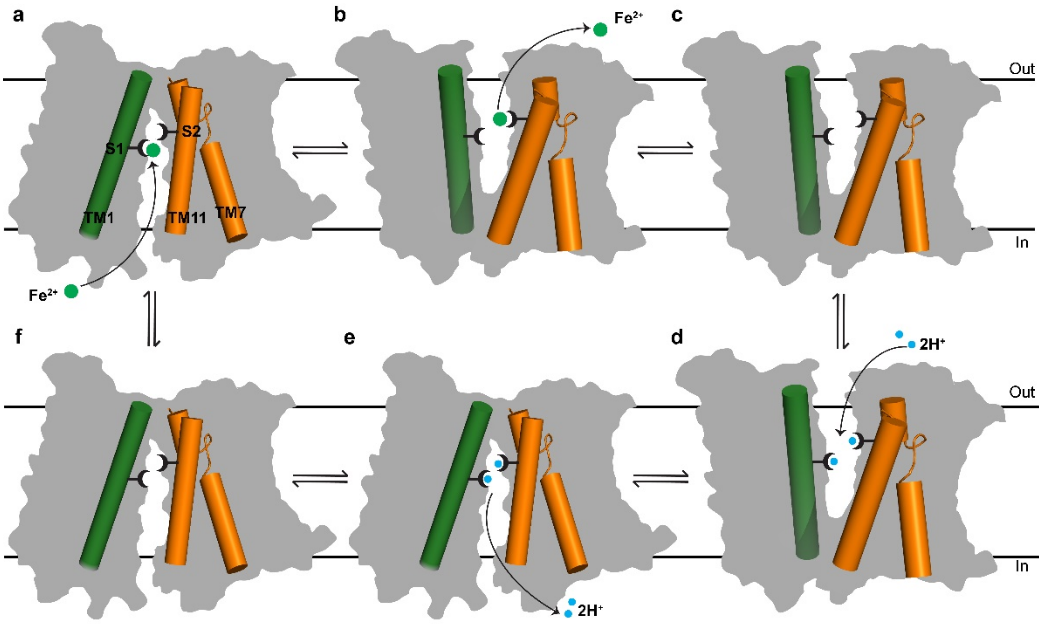

Supplementary Figure 9. Proposed mechanism of ion transport. a-f. Proposed conformational changes of Fpn during the transport cycle. A cytosolic Fe^{2+} ion (green sphere) binds to either the S1 or S2 sites (drawn as forks) of a Fpn at the inward-facing conformation (**a**); Fpn then switches to the outward-facing conformation to allow the bound Fe^{2+} to escape to the extracellular side (**b-c**). Two protons (blue spheres) bind to the S1 and S2 sites in an outward-facing conformation of Fpn (**c-d**) and enter the cytosol when Fpn returns to the inward-facing conformation (**e-f**).

Supplementary Table 1 | Summary of cryo-EM data collection, processing and structure refinement.

Protein	TsFpn-Fab	TsFpn-Hepcidin-Fab
**Cryo-EM Data Collection**		
Voltage (kV)	300	300
Magnification (x)	105,000	105,000
Pixel Size (Å)	1.114	1.114
Electron exposure (e-/Å^2/frame)	1.56	1.56
Defocus range (μm)	[-2.0, -1.2]	[-2.0, -1.2]
Number of image stacks	1838	1838
Number of frames per stack	32	32
**Cryo-EM Data Processing**		
Initial number of particles	946,473	540,037
Final number of particles	215,752	214,136
Symmetry imposed	C1	C1
Map sharpening B factor (Å^2)	-100	-308
Map resolution (Å)	3.0	3.4
Map resolution range (Å)	2.9-3.7	3.2-4.0
FSC threshold	0.143	0.143
**Model Refinement**		
Number of amino acids	847	846
Total non-hydrogen atoms	5392	5395
Average B factor (Å^2)	49.83	178.25
Bond length r.m.s.d. (Å)	0.005	0.011
Bond angle r.m.s.d. (°)	0.814	1.321
Ranmachandran Plot		
Favored (%)	92.65	77.76
Allowed (%)	7.35	19.95
Outliers (%)	0.00	2.28
Rotamer outliers (%)	0.28	2.52
EMRinger Score	2.56	1.18

Supplementary Table 2 | The rate of uptake and normalized intensity in transport assays.

Sample	Rate of uptake ($\times 10^{-2}$ min^{-1})		Normalized intensity at 400 s (%)		n
	Average	s.e.m.	Average	s.e.m.	
No protein (100 μM Fe^{2+})	2.18	0.22	93.07	0.63	3
Fe^{2+} 1 μM	5.12	0.24	86.45	0.32	4
Fe^{2+} 3 μM	8.27	0.48	81.00	0.21	3
Fe^{2+} 10 μM	9.68	0.29	78.37	0.67	3
Fe^{2+} 20 μM	10.46	0.68	75.09	0.65	3
Fe^{2+} 50 μM	11.31	0.33	70.46	0.79	3
Fe^{2+} 100 μM	15.47	1.17	64.70	1.56	3
No protein (500 μM Co^{2+})	0.47	0.18	98.53	0.55	3
Co^{2+} 1 μM	1.66	0.20	93.29	1.27	4
Co^{2+} 5 μM	3.88	0.39	88.33	1.00	4
Co^{2+} 20 μM	8.72	0.93	83.39	0.43	4
Co^{2+} 100 μM	10.58	0.57	74.75	0.72	3
Co^{2+} 300 μM	11.39	1.19	73.27	0.37	3
Co^{2+} 500 μM	12.41	0.27	63.16	0.56	3
No protein 2 mM Ca^{2+}	1.03	0.14	96.68	0.17	3
TsFpn No Ca^{2+}	12.41	0.27	63.16	0.56	3
TsFpn 2 mM Ca^{2+}	11.60	0.71	64.34	1.17	3
HsFpn No Ca^{2+}	9.65	0.33	66.88	1.00	3
HsFpn 2 mM Ca^{2+}	10.30	0.69	64.29	0.76	3
No protein (500 μM Co^{2+})	0.47	0.18	98.53	0.55	3
Fab (o) *	5.13	0.02	82.73	0.70	3
Fab (i)/(o) *	1.61	0.28	93.55	0.60	3
Hepcidin (o) *	5.27	0.67	80.85	0.43	3
Hepcidin (i)/(o) *	4.32	0.02	87.70	0.34	3
Fab (o) + Hepcidin (o) *	3.17	0.39	88.39	0.59	3
No inhibitor *	10.58	0.57	74.75	0.72	3

No protein 7.5(i)/8.5(o)	0.91	0.24	96.60	0.87	3
WT 7.5(i)/6.5(o)	9.67	0.55	79.98	0.73	3
WT 7.5(i)/7.0(o)	11.54	1.04	69.01	0.65	3
WT 7.5(i)/7.5(o)	12.41	0.27	63.16	0.56	3
WT 7.5(i)/8.0(o)	28.36	1.95	38.82	1.60	3
WT 7.5(i)/8.5(o)	53.78	3.72	27.28	0.59	3
WT 8.5(i)/8.5(o)	20.38	0.57	51.49	0.59	3
S1 7.5(i)/7.5(o)	10.27	0.15	61.27	1.87	3
S1 7.5(i)/8.5(o)	17.41	0.72	51.51	0.41	3
S2 7.5(i)/7.5(o)	6.71	0.57	79.20	1.75	3
S2 7.5(i)/8.5(o)	11.65	0.14	73.65	1.18	3
KCl(i)/KCl(o)	12.56	0.57	67.57	0.65	3
NaCl(i)/NaCl(o)	12.41	0.27	63.16	0.56	3
K-Gluconate(i)/K-Gluconate(o)	12.25	0.47	71.38	1.01	3
WT +120 mV	13.74	1.03	64.52	0.95	3
WT 0 mV	12.56	0.57	67.57	0.65	3
WT -120 mV	13.22	1.44	66.75	0.95	3
S1 -120 mV	29.28	0.41	34.18	1.75	3
S1 0 mV	12.25	1.08	64.87	1.36	3
S1 +120 mV	8.43	0.70	72.39	0.54	3
S2 -120 mV	12.92	0.70	63.94	1.22	3
S2 0 mV	5.97	0.15	78.53	1.20	3
S2 +120 mV	3.52	0.20	83.75	0.70	3
No protein pyranine	4.70	0.56	81.30	0.65	3
WT pyranine	40.02	1.99	26.19	1.40	3
S1 pyranine	34.67	0.25	32.10	1.03	3
S2 pyranine	7.62	1.25	62.68	2.83	3
S1 no hepcidin	10.27	0.15	61.27	1.87	3

S1 hepcidin	7.34	0.86	75.70	1.76	3
S2 no hepcidin	6.71	0.57	79.20	1.75	3
S2 hepcidin	6.71	0.73	79.89	1.45	3
WT no hepcidin	12.41	0.27	63.16	0.56	3
WT hepcidin	8.14	0.71	76.35	2.21	3

The rate of uptake and normalized intensity values were calculated as described in Methods. The errors are s.e.m. from at least three independent repeats (n).

The concentration of Co^{2+} is 500 μM unless otherwise specified.

* The concentration of Co^{2+} is 100 μM in these experiments.

Supplementary Table 3 | Primers used in this study.

Name	Sequence
D39A/H43A-f	5’- CCCTGTCCACTTGGGGCGCCCGTATGTGGGCCTTCGCTGTGTCCGTGTTC -3’
D39A/H43A-r	5’- GAACACGGACACAGCGAAGGCCCACATACGGGCGCCCCAAGTGGACAGGG -3’
C326A-f	5’- CCGTCCTGGGTTTCGACGCCATCACCACCGGTTACGC -3’
C326A-r	5’- GCGTAACCGGTGGTGATGGCGTCGAAACCCAGGACGG -3’
H508A-f	5’- CCTCCTGGACCTCCTGGCCTTCATCATGGTCATC -3’
H508A-r	5’- GATGACCATGATGAAGGCCAGGAGGTCCAGGAGG -3’

Reference

1 Ren, Z. *et al.* Structure of an EIIC sugar transporter trapped in an inward-facing conformation. *Proceedings of the National Academy of Sciences of the United States of America* **115**, 5962-5967, doi:10.1073/pnas.1800647115 (2018).

2 Bai, Y. *et al.* X-ray structure of a mammalian stearoyl-CoA desaturase. *Nature* **524**, 252-256, doi:10.1038/nature14549 (2015).

3 Sievers, F. *et al.* Fast, scalable generation of high-quality protein multiple sequence alignments using Clustal Omega. *Molecular systems biology* **7**, 539-539, doi:10.1038/msb.2011.75 (2011).

4 Robert, X. & Gouet, P. Deciphering key features in protein structures with the new ENDscript server. *Nucleic Acids Research* **42**, W320-W324, doi:10.1093/nar/gku316 (2014).

5 Smart, O. S., Neduvelil, J. G., Wang, X., Wallace, B. A. & Sansom, M. S. P. HOLE: A program for the analysis of the pore dimensions of ion channel structural models. *Journal of Molecular Graphics* **14**, 354-360, doi:https://doi.org/10.1016/S0263-7855(97)00009-X (1996).
